# Preventing complicated transseptal puncture with intracardiac echocardiography: case report

**DOI:** 10.1186/1476-7120-3-5

**Published:** 2005-03-01

**Authors:** Tchavdar Nikolov Shalganov, Dora Paprika, Sarolta Borbás, András Temesvári, Tamás Szili-Török

**Affiliations:** 1 Gottsegen György Hungarian Institute of Cardiology Haller utca 29, H-1096, Budapest, Hungary

## Abstract

**Background:**

Recently, intracardiac echocardiography emerged as a useful tool in the electrophysiology laboratories for guiding transseptal left heart catheterizations, for avoiding thromboembolic and mechanical complications and assessing the ablation lesions characteristics. Although the value of ICE is well known, it is not a universal tool for achieving uncomplicated access to the left atrium. We present a case in which ICE led to interruption of a transseptal procedure because several risk factors for mechanical complications were revealed.

**Case presentation:**

A case of a patient with paroxysmal atrial fibrillation and atrial flutter, and distorted intracardiac anatomy is presented. Intracardiac echocardiography showed a small oval fossa abouting to an enlarged aorta anteriorly. A very small distance from the interatrial septum to the left atrial free wall was seen. The latter two conditions were predisposing to a complicated transseptal puncture. According to fluoroscopy the transseptal needle had a correct position, but the intracardiac echo image showed that it was actually pointing towards the aortic root and most importantly, that it was virtually impossible to stabilize it in the fossa itself. Based on intracardiac echo findings a decision was made to limit the procedure only to ablation of the cavotricuspid isthmus and not to proceed further so as to avoid complications.

**Conclusion:**

This case report illustrates the usefulness of the intracardiac echocardiography in preventing serious or even fatal complications in transseptal procedures when the cardiac anatomy is unusual or distorted. It also helps to understand the possible mechanisms of mechanical complications in cases where fluoroscopic images are apparently normal.

## Background

Since the advent of ICE in the electrophysiology practice it proved its value in guiding transseptal procedures with providing an extra safety margin for the patients. The possibility to visualize the oval fossa, the LA free wall and the aortic root helps in preventing mechanical complications. ICE can visualize also intracardiac thrombus and spontaneous echocontrast, which is helpful in avoiding thromboembolic complications. Although the value of ICE is well known, it is rather hard to admit that it is a universal tool for achieving uncomplicated access to the left atrium. The aim of this case presentation is to show that ICE can lead to interruption of a transseptal procedure due to the presence of risk factors for mechanical complications when the fluoroscopic image is seemingly satisfying.

## Case presentation

**Figure 1:**
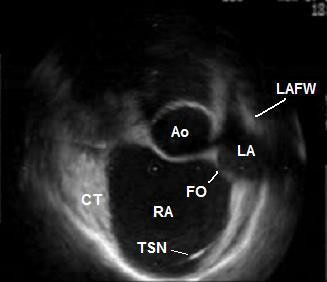
ICE during atrial diastole before the jump of the transseptal needle into the oval fossa. In this patient the LA cavity has a crescent-like shape at this time point of the cardiac cycle. Ao – non-coronary sinus of the aorta; CT – terminal crest; FO – oval fossa; LA – left atrium; LAFW – left atrial free wall; RA – right atrium; TSN – transseptal needle.

**Figure 2:**
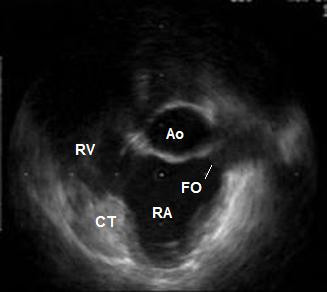
ICE during atrial systole before the jump of the transseptal needle into the oval fossa. The LA cavity is virtually missing at this time point of the cardiac cycle. Ao – non-coronary sinus of the aorta; CT – terminal crest; FO – oval fossa; RA – right atrium; RV – right ventricle.

**Figure 3:**
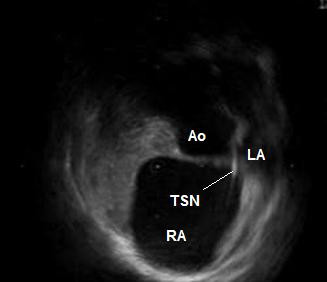
ICE after the jump of the transseptal needle into the oval fossa. The needle tip points towards the aorta. Ao – non-coronary sinus of the aorta; LA – left atrium; RA – right atrium; TSN – transseptal needle.

A seventy-year-old male patient with paroxysmal atrial fibrillation and atrial flutter, and concomitant arterial hypertension was referred to our institution for LA circumferential ablation. The preprocedural TEE described an aneurysm of the IAS (See Additional file 1: TEE.mpeg for the TEE), however the left heart catheterization was deemed feasible. ICE performed at the electrophysiology laboratory (catheter Ultra ICE 9900, 9 MHz, EP Technologies, Boston Scientific Corp., San Jose, CA, USA connected to a console ClearView Ultra, Boston Scientific Corp., San Jose, CA, USA) as a routine part of the transseptal puncture showed more detailed picture of the intracardiac anatomy – dilated aortic root, enlarged right atrium displacing the IAS towards the LA free wall, and small oval fossa with flapping motion of the fossa ovalis membrane. The distance between the IAS and the LA free wall during the atrial diastole was small (Fig. 1). During the atrial contraction the LA cavity appeared almost obliterated and the fossa ovalis membrane virtually touched the LA free wall, thus making the transseptal puncture potentially dangerous (Fig. 2) (See Additional file 2: ICE1.mpeg for the unusual intracardiac anatomy). The fossa itself was abouting anteriorly to the non-coronary aortic sinus of Valsalva and although the fluoroscopic position of the transseptal needle was apparently correct, ICE showed that it was always sliding to the junction between the IAS and the aorta and that the needle tip was pointing towards the aortic root. Several unsuccessful attempts were made to change its direction towards the oval fossa. That was also deemed a condition predisposing to complicated transseptal puncture (Fig. 3) (See Additional file 3: ICE2.mpeg for the sliding of the transseptal needle towards the aorta). During the procedure two echocardiographers, including the one, which had performed the TEE, re-evaluated the videotaped TEE and agreement was achieved that the initial interpretation of the TEE needed correction. Although the patient was symptomatic and the arrhythmia episodes could not be suppressed effectively with antiarrhythmic drugs, after considering all pros and cons we decided not to perform the left atrial procedure and thus to avoid serious and potentially fatal complications. Only a cavotricuspid isthmus ablation was done.

## Discussion

In the last years ICE emerged as a useful adjunctive tool in the field of interventional electrophysiology. It serves not only scientific purposes but practical issues as well. Its value for achieving successful and uncomplicated transseptal access to the LA cavity is well known [1-4]. However, it has not been used consistently for elucidating the possible mechanisms of mechanical complications during transseptal left heart procedures.

### ICE for transseptal puncture

In this case ICE showed that the aneurysm of the IAS observed during the TEE was actually the angulated continuity between the enlarged aortic root and the IAS. The echocardiographic orientations of ICE are sometimes clearly off axis in comparison to standard transesophageal echocardiographic views. Nevertheless, to our opinion ICE is superior in providing more detailed picture of the neighboring cardiac structures. Although useful in guiding transseptal catheterizations, TEE does not always provide complete avoidance of complications even in patients with normal hearts [5,6]. Furthermore, the oval fossa itself was small but its membrane nevertheless showed bidirectional flapping motion. This suggests that ICE and TEE images are indeed no equivalent. As the fossa was abouting the aortic root this flapping motion was located in the angle between the two structures and had probably given rise to the false impression of an aneurysm. Additionally, very small distance between the oval fossa and the LA free wall was also observed. In such circumstances the risk of puncturing the left atrial free wall is prominent even if the puncture of the oval fossa itself is uncomplicated [2,3]. Usually ICE-guided redirection of the transseptal needle is all that is needed for achieving uncomplicated access to the LA in such cases. In the case presented there was virtually no distance between the IAS and the LA free wall during the atrial systole, so such a maneuver would not be of help. Also it was not possible to achieve a stable position of the transseptal needle in the fossa itself. It always slided and pointed to the aorta. This means that even if the distance from the oval fossa to the LA free wall was large enough the puncture would be complicated. To our opinion this is one possible explanation for the rare instances of inadvertent puncturing of the aorta when the fluoroscopic images appear to be completely satisfying.

### ICE for avoidance of other complications

EP procedures are relatively safe procedures and have low complication rate. One of the most frequent complications is related to cardiac wall perforation with consequent pericardial effusion and tamponade. ICE, especially the one with phased-array transducer (deeper penetration) allows continuous monitoring of the pericardial space during EP procedures. This permits prompt detection of a pericardial effusion and an immediate guidance of a therapeutic puncture. Phased-array transducers are equipped with Doppler capabilities allowing assessment of the pulmonary venous flow pattern after pulmonary vein ablation on top of diameter measurements to exclude pulmonary vein stenosis, which is the most important complication of this procedure. Monitoring microbubble formation during RF energy application allows prevention of pulmonary vein stenosis [7]. ICE also allows detection of intracardiac thrombi during the procedures, especially during left-sided ablations [8].

## Conclusion

We strongly believe that this case is a good illustration of the usefulness of the ICE in the electrophysiology field and further enhances its value as a tool for avoiding complications when the intracardiac anatomy is unusual or distorted. As the number of transseptal procedures in the electrophysiology laboratories all over the world is steeply growing ICE definitely has the potential to become a routine at least in those institutions with large volume of left atrial procedures.

## List of abbreviations

EP – electrophysiologic

IAS – interatrial septum

ICE – intracardiac echocardiography

LA – left atrium; left atrial

TEE – transesophageal echocardiography

## Supplementary Material

Additional File 1 Preprocedural transesophageal echocardiography. The irregular oval-shaped structure at the center of the screen is the aortic root. At a certain moment one can see at its upper part the ostium and the most proximal part of the right coronary artery. Below is situated the left atrium and to the left – the right atrium. The oval fossa is in between. Click here for file

Additional File 2 Intracardiac echocardiography, showing distorted intracardiac anatomy. The oval shape in the center of the screen is the non-coronary sinus of Valsalva. Below is the right atrium at the bottom of which the transseptal needle is clearly visible. The prominent muscular structure in the left-hand part of the image is the terminal crest. The membrane of the oval fossa, adjacent to the right-hand part of the non-coronary aortic sinus shows bidirectional flapping motion. During the atrial contraction the cavity of the left atrium virtually disappears. Click here for file

Additional File 3 Intracardiac echocardiography showing sliding of the needle towards the aorta. The transseptal needle is already in the oval fossa with its tip pointing to the aorta. This is especially clearly visible after a premature beat. Click here for file
